# The 2025–2030 US Dietary Guidelines: an analysis of scientific integrity and global health governance

**DOI:** 10.1016/j.lana.2026.101402

**Published:** 2026-02-14

**Authors:** Felipe Silva Neves, Eduardo Augusto Fernandes Nilson, Larissa Loures Mendes, Neha Khandpur, Marion Nestle

**Affiliations:** a Group for Studies, Research and Practices on Food Environment and Health (GEPPAAS), Department of Nutrition, School of Nursing, Federal University of Minas Gerais (UFMG), Belo Horizonte, Brazil; b Department of Nutrition, Institute of Biological Sciences (ICB), Federal University of Juiz de Fora (UFJF), Juiz de Fora, Brazil; c Oswaldo Cruz Foundation (FIOCRUZ), Brasília, Brazil; d Department of Nutrition, School of Nursing, Federal University of Minas Gerais (UFMG), Belo Horizonte, Brazil; e Division of Human Nutrition and Health, Wageningen University & Research (WUR), Wageningen, Netherlands; f New York University (NYU), New York, USA

The United States (US) has recently released the Dietary Guidelines for Americans 2025–2030. While the policy introduces sound recommendations for vegetables, fruits, and whole grains, and limits added sugars and ultra-processed foods (UPFs) (termed “highly processed foods” within the text), it fails to reflect the contemporary scientific consensus by prioritising animal proteins, animal fats, and full-fat dairy products.^1^ Additionally, this political position follows a supplementary institutional report that dismisses previous efforts to include health equity and social determinants in the empirical evidence base, labelling such integration a “methodological deficiency”.^2^ Consequently, these guidelines depart from the international standards required for non-communicable diseases (NCDs) prevention.^3^^,^^4^ Given US normative influence, this regression legitimises corporate interests, threatening transnational health governance and food and nutrition security.

The inherent contradiction within the 2025–2030 guidelines is profound. By promoting animal-source proteins and full-fat dairy, the document proposes a dietary pattern fundamentally inconsistent with its own goal of limiting saturated fat intake below 10% of total calories.^1^ This internal incoherence reflects decision-making that continues to prioritise the economic interests of specific industrial sectors over NCDs prevention.^5^ The paradoxical nature of the guidelines is evidenced by the reliance on an anachronistic visual communication tool. While the inclusion of processing-based terminology is a progressive step, the reintroduction of a hierarchical food pyramid model represents a semiotic retreat into a reductionist era of public health. This abstraction fails to capture the complexity of modern food systems or the distinction between food types and the extent of industrial processing.^3^ While the international community moves towards representations emphasising fresh foods and the social context of eating, the US return to a pyramid isolates nutrients from the food matrix.^6^

In sharp contrast, the Dietary Guidelines for the Brazilian Population remains the gold standard for food and nutrition frameworks.^6^ Published in 2014, the Brazilian approach introduced an epistemological shift away from the dominant reductionist paradigm by moving dietary advice towards the degree and purpose of industrial food processing. This strategy, facilitated by the Nova classification system, acknowledges that industrial alterations to food matrices have wide-reaching implications for biological integrity, metabolic health, social structures, and environmental sustainability. As Table 1 illustrates, the contrast between the US and the Brazilian standards is defined by their diverging methodological and conceptual foundations. By prioritising an adequate and healthy diet centred on fresh and minimally processed foods, Brazil provides a robust template for addressing the interactions between human biology, cultural identity, and planetary health, achieving maximum scores across metrics of public health and sustainability.^3^^,^^4^^,^^7^

**Table 1:** Conceptual and methodological comparison between the 2025–2030 US Dietary Guidelines and the Dietary Guidelines for the Brazilian Population.

Feature	Dietary Guidelines for Americans 2025–2030^1^	Dietary Guidelines for the Brazilian population^6^
Guiding paradigm	Focus on nutrient density and individual responsibility; health is framed as a matter of personal choice and moral deficit.	Multidimensional approach integrating biological, social, and environmental health; focus on food systems and collective well-being.
Scientific integrity	Supplementary report authored by experts with documented conflicts of interest with the beef, dairy, and food industries.	Independent process led by academic researchers, strictly free from commercial influence and industry sponsorship.
Classification system	Traditional food groups with an absence of technical criteria for industrial processing; focus remains on isolated nutrients.	Nova classification categorising foods by the degree and purpose of industrial processing (fresh and minimally processed foods, culinary ingredients, processed foods, and UPFs).
Visual communication	Reintroduction of an anachronistic hierarchical food pyramid model, representing a semiotic retreat into reductionism.	Rejection of the pyramid in favour of food-based representations that emphasise meals and the social context of eating.
Core recommendations	Prioritisation of animal proteins and full-fat dairy; selective recommendations against processed products.	Dietary foundation of fresh, plant-based foods and the categorical avoidance of UPFs.
Saturated fat management	Mathematical paradox between a 10% intake limit and the promotion of animal fats; absence of guidance on unsaturated fat substitution.	Achieved through patterns based on fresh foods; explicit emphasis on replacing animal fats and UPFs with plant-based oils and whole foods.
Environmental sustainability	Omission of the climate crisis and planetary boundaries from the policy framework; silence on the environmental impact of livestock.	Sustainability as a core principle; promotion of biodiverse, just, and resilient food systems that respect planetary limits.
Equity and determinants	Rejection of the “health equity lens”; social and environmental determinants dismissed as a “methodological deficiency”.	Structural pillars: integration of social justice, social determinants of health, and the promotion of food sovereignty.
Global influence and sovereignty	Functions as a permissive framework that dilutes the narrative on food sovereignty and serves as a scientific alibi for industrial actors.	A paradigm of regulatory sovereignty; provides the conceptual framework for pioneering policies like warning labels and fiscal measures.

UPFs = ultra-processed foods.

The conceptual divergence between these frameworks reflects a broader tension between public health principles and the narrative of personal responsibility. By rejecting social and environmental determinants, the 2025–2030 US guidelines shift the entire burden of health onto individual choice, ignoring the structural barriers defining the food environment.^2^^,^^8^ The reintroduction of individual responsibility as the central axis of food policy is an ideological framework that converts systemic failures into individual moral deficits, thereby legitimising state regulatory inaction.^5^^,^^9^ In an era where NCDs prevention requires robust environmental and policy interventions, the US return to a personal responsibility framework represents a dangerous abdication of the state-led public health mandate.^4^

The erosion of scientific integrity within the US policy framework is a manifestation of the commercial determinants of health.^5^ The formulation of these guidelines suggests a clear case of corporate capture. While official political discourse promises to “Make America Healthy Again” by addressing corporate influence, the supplementary scientific report was authored by experts with documented conflicts of interest with the beef, dairy, and food industries.^1^^,^^2^ These commercial interests have effectively undermined the promotion of an adequate and healthy diet. Reversing this trend requires decisive state-led interventions beyond individual choice, necessitating robust policies to restrict UPF production and structural reforms to address the corporate actors dominating global supply.^4^^,^^10^

The failure of the 2025–2030 US guidelines to address the environmental dimensions of diet is negligent in an era of ecological instability. While the international community aligns with the EAT-Lancet 2.0 consensus, which emphasises that food systems must operate within planetary boundaries, the US guidelines remain silent on the climate crisis.^8^ Transitioning towards plant-forward diets is a foundational requirement for mitigating the environmental degradation caused by intensive livestock systems. The omission of these factors ignores the reality of the Global Syndemic, in which obesity, undernutrition, and climate change are interconnected pandemics driven by the same food system failures.^9^ By failing to address environmental impacts, the US promotes a model of consumption linked to planetary health degradation, further endangering global food and nutrition security.

The axis of scientific integrity has shifted to the Global South, where Latin American nations—including Brazil, Chile, Colombia, Mexico, Peru, and Uruguay—uphold food systems that are socially just and environmentally sustainable. These countries have pioneered transformative interventions, such as Brazil's focus on food processing, Chile's warning labels, and UPF taxation in Mexico and Colombia.^4^ The political and economic power of the US increases the likelihood that its guidelines will be leveraged by transnational corporations to dismantle these regulations. In international bodies like the *Codex Alimentarius*, the US framework provides a scientific alibi for industrial actors to dispute sovereign policies, framing evidence-based regulations as barriers to trade. This reflects documented precedents, such as the use of US policy to circumvent international protections for breastfeeding, illustrating how domestic guidelines can function as instruments to impede global health progress.^5^

Ultimately, the 2025–2030 US Dietary Guidelines do not represent a legitimate departure from scientific progress, but a case of corporate capture with direct implications for national and global morbidity. The World Health Organization, the Food and Agriculture Organization of the United Nations, and the global public health community must act decisively to protect the integrity of dietary guidelines against the corporate concessions represented in the US guidelines, which dismiss established evidence on the health impacts of food processing and social determinants. Transnational health governance must be insulated from the influence of corporations that seek to undermine public health for private gain.^5^^,^^10^ Resisting the global influence of this flawed framework is essential to ensure that future generations have access to an adequate and healthy diet that respects both cultural heritage and planetary limits.

The leadership vacuum created by the US concessions offers a pivotal opportunity for the Global South to redefine public health governance. Latin American nations, supported by regional networks such as the Latin American Inter-institutional Network for Technical Cooperation on Food Environments and the Prevention of NCDs, are already demonstrating the efficacy of science-based, conflict-of-interest-free leadership. Safeguarding global health now requires fostering cross-regional collaborations, securing independent funding, and consolidating Brazil and the region as the pioneers of food system transformation. The era of corporate concessions has ended; the era of evidence-based leadership has begun.

## Contributors

FSN conceptualised the study. FSN, EAFN, LLM, NK, and MN performed the formal analysis. FSN drafted the original manuscript. EAFN, LLM, NK, and MN provided critical revisions and edited the manuscript. All authors had full access to all the data in the study and had final responsibility for the decision to submit for publication.

## Declaration of interests

The authors declare no competing interests.
